# Draft Genome Sequence of the Nicotinate-Metabolizing Soil Bacterium *Bacillus niacini* DSM 2923

**DOI:** 10.1128/genomeA.01251-14

**Published:** 2014-12-04

**Authors:** Zachary H. Harvey, Mark J. Snider

**Affiliations:** Department of Chemistry, The College of Wooster, Wooster, Ohio, USA

## Abstract

*Bacillus niacini* is a member of a small yet diverse group of bacteria able to catabolize nicotinic acid. We report here the availability of a draft genome for *B. niacini*, which we will use to understand the evolution of its namesake phenotype, which appears to be unique among the species in its phylogenetic neighborhood.

## GENOME ANNOUNCEMENT

*N*-Heterocyclic aromatic compounds, common constituents of pharmaceuticals and herbicides, constitute environmental contaminants of emerging concern due to their water solubility and carcinogenicity. A number of species are known to subsist on *N-*heterocycles as their sole source of carbon (1, 2). *Bacillus niacini* is among these, having been shown to survive on nicotinate (3, 4). While two enzymes and their cofactors (5, 6) have been putatively identified, the genes that encode them and the remainder of the relevant cluster are unknown.

The *B. niacini* genome was sequenced at ACGT, Inc. (Wheeling, IL) using the Illumina MiSeq platform, resulting in >9 million high-quality (>Q30) reads derived from paired-end and mate-paired libraries after adapter trimming and filtering. We chose to assemble and finish the *B. niacini* genome using the Mix software package (7), as only distantly related reference genomes (8, 9) were available. Mix uses multiple draft assemblies to remove redundant or low-coverage contigs, as well as to merge overlapping ones. Both sequence libraries were assembled using Velvet (10), ABySS (11), and SOAP*denovo*2 (12) and then combined using Mix to produce a finished genome sequence consisting of 447 contigs with a total length of >6 Mb and a G+C content of 38%. This assembly was annotated using the Rapid Annotations with Subsystems Technology (RAST) server (13), which annotated 5,904 protein-coding sequences, as well as 204 RNAs.

While the G+C content of the *B. niacini* genome is consistent with low-GC Gram-positive bacteria, the genome size is much larger than that of other closely related species (14, 15). Using a subtractive genomics approach, we were able to identify a novel cluster of genes not found in other *Bacillus* species (16). This cluster segregated into a single contig 12,852 bp in length, which contains 13 genes, including two putative regulatory genes. While many of these genes appear novel to *B. niacini*, two of them possess notable sequence similarities to *Pseudomonas putida nicF* and *nicE*, both of which are involved in nicotinate catabolism (1). Following an analysis of the predicted protein architecture, we were able to identify a number of open reading frames (ORFs) with features consistent with previous observations (5, 6). Among these, a pair of putative molybdenum-dependent oxidoreductases is present, along with at least one iron-sulfur cluster-binding protein. The putative regulatory genes belong to the TetR and IclR families, possibly acting as activators (17) and repressors (18), respectively. While it is unclear if these regulatory proteins act on the identified gene cluster, we expect that such an operator structure would be present based on the observation that nicotinic acid and its metabolites induce pathway expression (4, 6, 19). While two genes appear biochemically similar to genes in phenotypically equivalent clusters, the majority are radically different in both their predicted biochemistry and codon usage (1). These observations present a conflicting picture of how the cluster arose in *Bacillus* and may suggest that its evolution is an interplay of both horizontal gene transfer and convergent evolutionary events.

### Nucleotide sequence accession number.

This draft genome sequence has been deposited in GenBank under the accession no. JRYQ00000000.
